# Blurred interface induced control of electrical transport properties in Josephson junctions

**DOI:** 10.1038/s41598-024-68285-y

**Published:** 2024-07-27

**Authors:** Junling Qiu, Huihui Sun, Chuanbing Han, Xiaodong Ding, Bo Zhao, Shuya Wang, Lixin Wang, Zheng Shan

**Affiliations:** Laboratory for Advanced Computing and Intelligence Engineering, Zhengzhou, 450001 China

**Keywords:** Structural materials, Theory and computation

## Abstract

The interfacial microstructures of Josephson junctions are vital for understanding the microscopic mechanism to improve the performance of superconducting qubits further. However, there remain significant concerns about well understanding the correlation between atomic structures and electrical behaviors. Here, we propose a new method to define the interface of the barrier in Josephson junctions, and investigate the factors that affect the electrical properties of junctions using material analysis techniques and first principles. We find that the aluminium–oxygen ratio of the interface contributes greatly to the electrical properties of junctions, which is consistent with the conclusions obtained by utilizing the generative adversarial network for data augmentation. When the aluminium–oxygen ratio of the interface is 0.67–1.1, the model exhibits a lower barrier height and better electrical properties of the junction. Moreover, when the thickness of the barrier is fixed, the impact of the aluminium–oxygen ratio becomes prominent. A detailed analysis of Josephson junctions using a microscopic model has led to identifying of process defects that can enhance the yield rate of chips. It has a great boost for determining the relationship between microstructures and macroscopic performances.

## Introduction

Josephson junction (JJ) is a crucial component of the superconducting quantum chip, which provides a lossless nonlinear inductor to control the qubit’s frequency by integrating the effective total capacitance of the qubit circuit^1^. Al/$\mathrm AlO_x$/Al junctions fulfill this function with superconducting quantum computers^2,3^. Even though recent progress has enabled multiqubit designs to exhibit coherence times on the order of hundreds microseconds, material quality and interface structures still restrict the device’s performance^4–9^. The presence of parasitic two-level defects is mainly attributed to the amorphous oxide in the interface of the material^10–12^, making the study of interfaces a hot issue.

Indeed, a great deal of research has been devoted to understanding and optimizing the microstructure of the interface on the performance of devices. It has been found that the tunneling current varies exponentially with the alumina thickness of the tunnel junction^13^. Eernest et al.^14^ proposed that the quality of the substrate–metal interface affects the electrical performance of the entire JJ. Fritz et al.^15^ obtained a flatter Al/$\mathrm AlO_x$ interface by optimizing the growth of the lower Al metal to improve the properties of the junction. Experimental characterization is an effective method to understand the structure and properties quickly, but limited by long cycles and materials themselves.

First-principles provide a powerful foundation for investigating the microscopic structures at interfaces, delivering high accuracy without material constraints. Cyster et al.^16^ calculated the electrical transport properties by non-equilibrium Green’s function (NEGF). They examined the influence of the density and stoichiometric fluctuations of $\mathrm Al_2O_3$ on the performance of JJ. Koberidze et al.^17^ studied the influence of six different Al/$\mathrm Al_2O_3$ interfaces on electron transport at the atomic scale, and the results showed that small changes in the atomic arrangement in the interface would lead to significant changes in the electron transport characteristics. Jung et al.^18^ proved that the qualitative consistency between computations and experiments is maintained when parameters from first-principles calculations are compared with fitted parameters from experimental data. Tea et al.^19^ used the parameters calculated by density functional theory (DFT) to test the accuracy of the barrier model. The complexity associated with experiments on amorphous interfaces hinders the advancement of a universal analytical model that can precisely predict interface phenomena^20^. Therefore, these studies mainly focused on the constructed models, with little integration with experiments.

Motivated to solve this conundrum, we applied generative adversarial network (GAN) to expand datasets for data augmentation. The foundations of the method rely on the results of electronic properties using DFT, and the statistical data from the barrier layers. GAN has previously been successfully applied to image generation^21,22^, photo editing^23,24^, video prediction^25,26^, and other data with continuity. Our method can have an impact on the research field by increasing the application field of GAN in discrete data or transport models.

In this work, we introduce a novel method to characterize the barrier interface of JJs, and explore the factors impacting its electrical properties through materials analysis techniques and first-principles studies. Combining the Al–O ratio of the interface with the electrical properties, the interface classification mode of the junction is supposed by the normal resistance. We find that the Al–O ratio greatly contributes to the electrical properties of the overall junction, which is consistent with the results predicted by using GAN to augment the model. When the Al–O ratio is between 0.67 and 1.1, the potential height is lower with better electrical properties. Moreover, when the thickness of the barrier is fixed, the impact of the Al–O ratio becomes prominent. Building a bridge between microscopic structure and macroscopic characteristics, with the study of key interfaces as the focal point, we can identify factors that influence the electrical properties in addition to thickness and junction area. This provides new insights about the effect of microstructure on the performance of superconducting quantum chips.

## Experiments

### Device model construction

**Figure 1:**
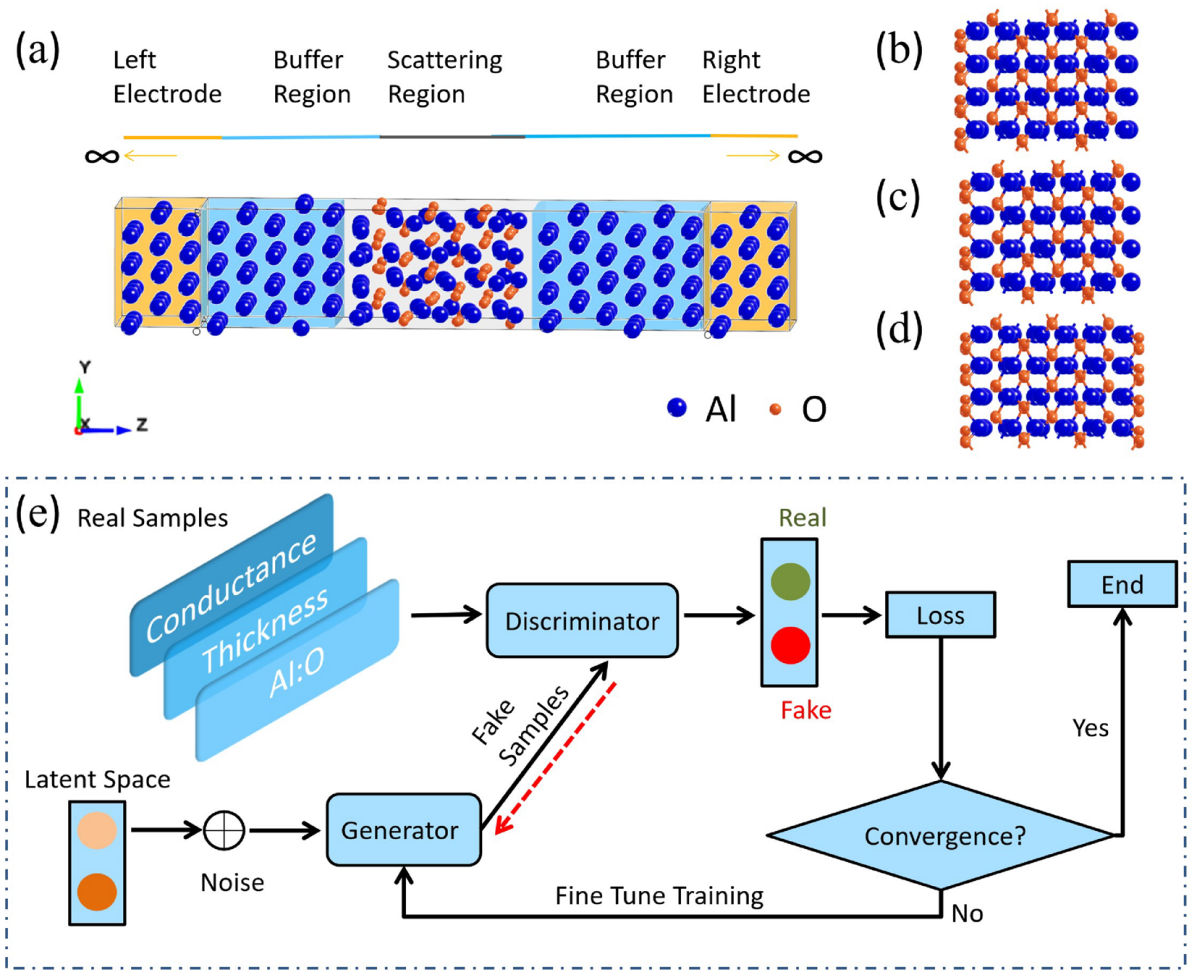
Schematic of the model. (**a**) Original model of Al/$\mathrm Al_2O_3$/Al Josephson junction with atomic two-port devices. (**b**–**d**) Atomic models of the outermost layers of $\mathrm Al_2O_3$ are O and 2Al, O and Al, O and O, respectively. The scattering region is composed of these three structures and corresponding different thicknesses, with a total of 36 models. (**e**) GAN model.

The model of JJ is constructed with Al/$\mathrm Al_2O_3$/Al three-layer structure. The atomic model can be divided into electrode regions, buffer regions and a central scattering region (as shown in Fig. 1) and the lattice constant of bulk aluminum is a = 4.0495Å. The buffer zones are made of the same material as the electrode zones, mainly to shield the electrodes from scattering effects. $\alpha$-$\mathrm Al_2O_3$ is adopted in the scattering region with lattice constants a = 4.759Å and c = 12.99Å. To compare with the manufacturing process, the material parameters are set under the lattice constants obtained by the experiments^20,27^. When constructing the initial models, we fixed the left and right electrodes and the outermost atoms of $\mathrm Al_2O_3$ in the center region are single-layer Al, double-layer Al and O, respectively^28–31^. Comprising three layers of Al atoms, the electrode region forms a semi-infinitely extending structure, accompanied by a six-layer Al buffer layer. Geometric optimization is performed with a projector-augmented wave method based on DFT^32–35^. Since the model is composed of different materials, to ensure the structural stability of the final model, we take different interface contact distances to calculate the single point energy, and finally take the model with the lowest energy as the best model.

Nanodcal^36^, based on DFT and NEGF, is used to calculate the quantum transport properties of the models^37^. The area of the junction is fixed by $0.84 \times 0.97~\hbox {nm}^{2}$^37,38^. The atomic structure of the model is shown in Fig. 1a–d, where different models of outer-most atomic types in the scattering region correspond to Fig. 1b–d, respectively. We construct different termination mode device models with thickness variations to characterize transport properties. The specific models are shown in the previous results^37,38^.

### Data augmentation model

With small datasets, it’s difficult to uncover the regularities among the data. Moreover, the time and capital costs of calculating models using DFT are very high. As an important member of deep learning, GAN was proposed by Goodfellow in 2014 based on the principles of zero-sum game and Nash equilibrium^39^, which is used to learn generative models from complex data. More datasets can be generated by GAN, and the loss function is iteratively calculated to ensure that it is within a certain error range until it converges. The GAN model consists of a generator and a discriminator, as shown in Fig. 1e. The generator is used to create data samples close to the real training data to fool the discriminator and act as an adversary. The loss function is defined to judge the success of the generator; that is, when the discriminator cannot identify whether the generated data is true or false, the generator succeeds. If the generator fails, the training process is iterated using the defined optimizer, as shown in Fig. 1e.

Our samples are derived from the Al–O ratio at the left and right interfaces of each model, the thickness of the barrier and the conductance calculated by NEGF^37^, which is shown in the input of Fig. 1e. The generator uses the stochastic gradient descent (SGD) method to create an optimizer, and the loss value during the training process is recorded. Mean squared error is used to create the loss function in the discriminator. After several iterations, the loss function converges to an accuracy of the order of 1E−3. The loss functions of the generator and discriminator oscillate at 0.25 after a short shock^40^, indicating the high quality of the generated data. This conclusion is also confirmed by comparison with the sample data.

Applying GAN to the augmentation of atomic structure models not only maintains the internal rules between data, but also saves the operation cost. In the sample data, it is expected that the generated data is as close as possible to the sample data, which fills the gap in the application of GAN in text data.

## Results and discussion

### The layered structure of the interface

Experimental studies show that key interfaces (including metal–insulator (MI) interface, substrate–metal (SM) interface), substrate loss, junction geometry and other factors affect the dielectric loss of the junction^1^, and then affect the coherence time of qubits. The characteristics of key interfaces, such as terminal mode, reconstruction and relaxation, can affect the quality of film interfaces^41^. The study about the microstructure of JJs and the key interfaces is largely decisive for the in-depth study and optimization of superconducting circuit performance.

**Figure 2:**
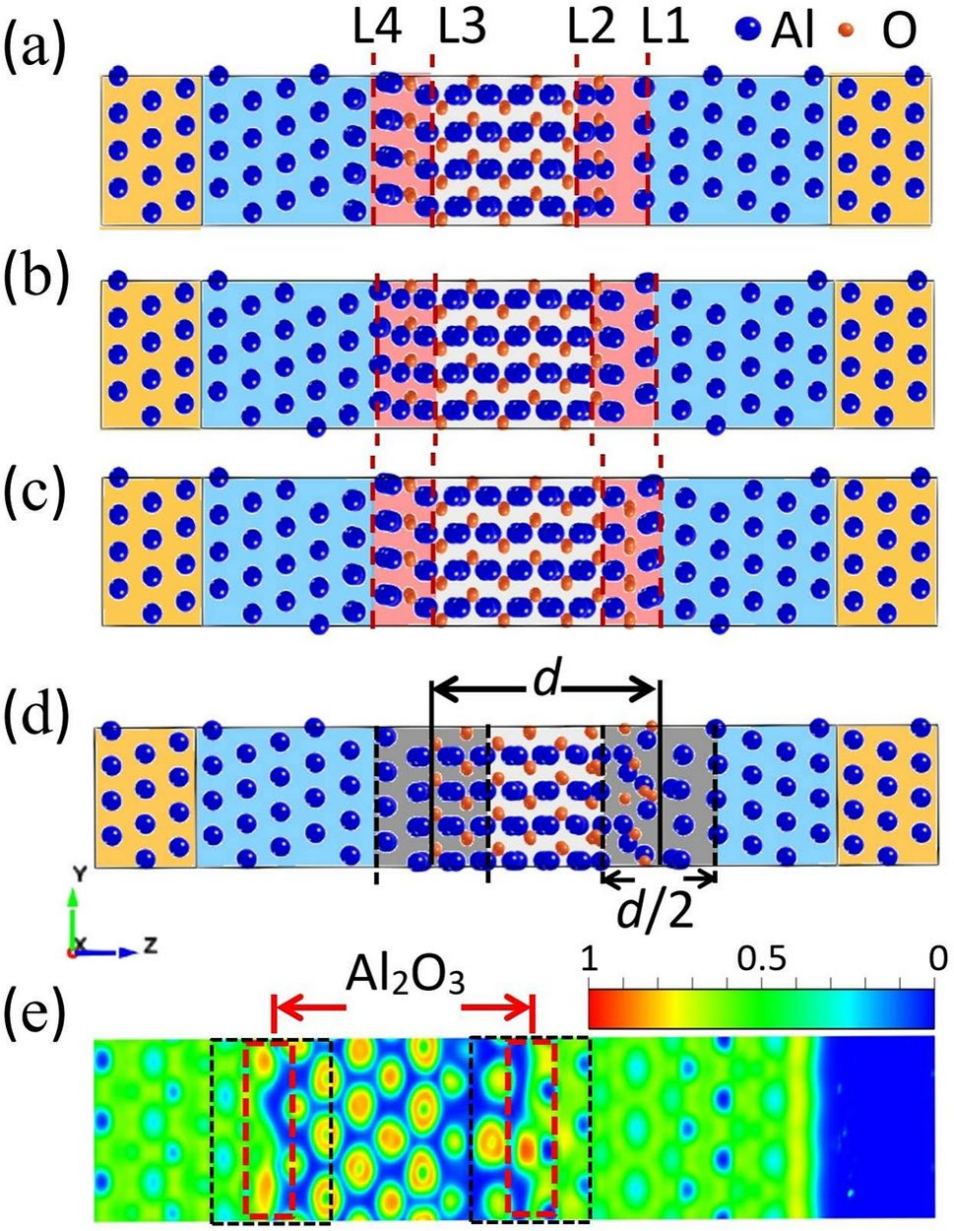
Interface partition models. (**a**–**c**) shows the layered models with the outermost atoms of O–Al, O–2Al, and O–O structures, respectively. (**d**) Interface division model by Cyster^42^. (**e**) ELF shows the unique bonding characteristics of metal-oxide. The red dashed box is the interface boundary defined in this paper, and the black dashed box is the interface boundary defined by Cyster^42^.

After the initial model relaxation, the atomic positions at the splice of the heterogeneous junctions are shifted, and the atomic structures are changed. Lattice changes will be found in the contact region between metal and oxide, and the chemical bonds of O in oxide and Al in metal will be recombined, resulting in a transition region between metal and oxide, called the interface. Changes in the microstructure, especially in the interface, directly affect the performance of JJ^42,43^. Therefore, it is necessary to define the interface further.

For example, the outermost atoms at the left interface of the barrier before relaxation are O atoms, as shown in Fig. 2a–c. The barriers are composed of Al, double Al, and O atoms as the outer atoms in the right interface, corresponding to O–Al terminal, O–2Al terminal and O–O terminal mode, respectively. According to the pair distribution function of amorphous $\mathrm Al_2O_3$, the probability of the distance between Al and O is the largest when the distance is around 2Å^44,45^, which is taken as the standard of the distance between the left and right interfaces. Taking Fig. 2a as an example, the distance between atoms on the left and right sides of L1 is 2.8Å, which is 22% larger than the distance between the other Al layers of the buffer region (2.3Å). The distance between atoms Al and O on the left and right sides of L2 is 1.04Å, which is 21% larger than the distance between the middle Al and O atomic layer (0.86Å). Similarly, there is an analogous rule between L3 and L4. The interface between the oxide layer and the metal layer of other models is determined similarly, and the Al–O atomic ratio in the interface layer is calculated (as shown in Table 1).

**Table 1:** Al–O ratios of different models after relaxation.

Interface classification	Atomic type of the interface	Al:O	
		Left interface	Right interface
O-dominated	Al–2Al	1:1	2:3
	2Al–Al	2:3	1:1
	O–2Al	1:1	2:3
	2Al–2Al	1:1	2:3
Al–O-balanced	Al–Al	4:3	4:3
	O–Al	5:3	1:1
	Al–O	1:1	5:3
	2Al–O	5:3	1:1
Al-dominated	O–O	5:3	5:3

In terms of the definition of the interface layer, there have been some studies^15,17,46^. Mei et al.^43^ determined the interface positions according to the change of the lattice spacing near the interface between bulk Al and $\mathrm Al_2O_3$ before and after relaxation. Due to the lattice mismatch, after model relaxation, there will be a lattice deformation in the interface between metal and oxide, and part of the oxide will move to the metal layer, resulting in a larger inter-atomic crystal spacing between Al and O in the contact surface than the crystal spacing in single crystal oxides. However, the thickness of the interface layer identified by this method contains $\mathrm Al_2O_3$ with a distance of more than 1 nm, then in the JJ fabricated by superconducting qubits, the total thickness of the oxide layer is only less than 2 nm. Furthermore, the method of dividing the interface layer by the change of lattice spacing is limited to the simulation model, and it cannot measure the lattice spacing of amorphous $\mathrm Al_2O_3$ in practice. Koberidze et al.^17^ used the variation interval of the projected density of states calculated by the model as the thickness of the interface layer, and determined the different thicknesses of the interface layer with different stacking methods. It is reasonable to determine the thickness of the interface layer from the simulation calculations perspective.

However, in the chips, there may be many ways of stacking or terminal modes in the barrier, and it is challenging to correlate with the experimental results directly. Cyster et al.^42^ mentioned another interface division method, where the thickness of the barrier layer was defined by the positions of the outermost oxygen atoms in the boundary oxide layers, and 1/2 of the thickness is used as the boundary, as shown in Fig. 2d. This definition can be applied in experimental representations and is a common method for interface partitioning. The difference in interface partitioning methods can be seen more clearly in the Electronic Localization Function (ELF). The transition of interfacial ions from metal Al to $\mathrm Al_2O_3$ shows the non-local continuous distribution and local island distribution characteristics^47^. This transition is not abrupt, but changes gradually along the vertical direction, as shown in Fig. 2e. The black dashed box is the interface layer defined by Cyster^42^, and the red dashed box is the interface region determined by our method. With Cyster’s method, the Al layer will be too much involved in the interface, which will affect the determination of the real roughness of the interface. And our method agrees well with the results of ELF.

The interface partition method proposed in this paper not only can observe the change of lattice spacing from the simulation aspect, but also is suitable for experimental characterizations. The redefinition of the interface layer is the first and important step to constructing the relationship between microstructure and macroscopic performance. Based on this method, the practical significance of the calculation results of the models is further studied.

### Interface classification

Both simulations and experiments have shown that the properties of JJs strongly depend on the interface of the Al$\_$based tunnel barrier. In simulation, much literature has studied the effects of the nature about junctions from different terminal modes. Starting from DFT, Koberidze et al.^17,48^ defined the terminal mode of $\mathrm Al_2O_3$ as Al terminal and O terminal according to the atomic positions, and found that the structural irregularities on the surface of the Al film near the interface made a significant contribution to lowering the barrier height. Shan et al.^37^ improved this method by adding double aluminum terminals to explore the relationship between the microstructure and electrical properties of JJs with more comprehensive atomic models, which has a guiding significance for the accurate control of the preparation of JJ.

Nevertheless, previous simulation studies mainly focused on the type of the outermost atoms in the barrier layer; it should be the multi-layer atoms of the critical interface that affect the barrier performance^48^. The characterization experiments show no such obvious boundary between the metal and the oxide in the interface. Ahn et al.^41^ experimentally verified that the terminal mode could be measured. They used time-of-flight scattering and recoiling spectrometry to obtain the scattering and recoil intensities from the exposed atoms in the first and second atomic layers of the alumina surface, and calculated the relative atomic concentration on the surface. The stacking order of Al and O in the surface was analyzed. This method is restricted by the limitations of the measuring equipment itself, and the accuracy of the calculated concentration is only 20%^41^. We believe that the types of terminal modes are certain, whether from the perspective of simulation or experimental characterization. However, due to the constraints of the process level, the interfaces of the barrier layer in the chip may be a mixture of various modes. To obtain a more accurate analysis of interface elements, we combine simulation models and experimental characterization to classify the interface characteristics based on the Al–O stoichiometric ratio.

**Figure 3:**
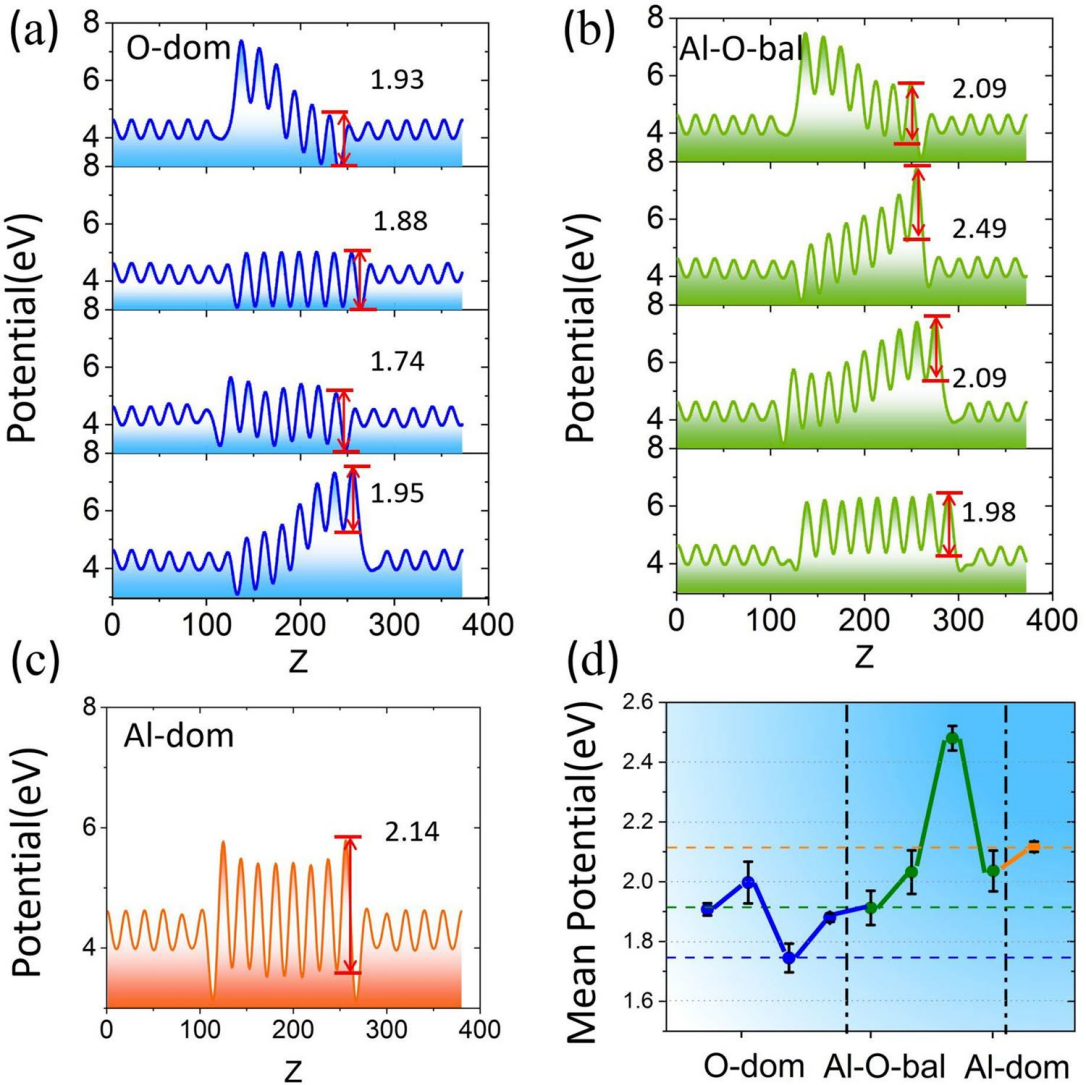
The potential of Al/$\mathrm Al_2O_3$/Al device along the transport direction with different interface classification modes. (**a**–**c**) The potential of the device of O-dom, Al–O-bal, and Al-dom, respectively. (**d**) Average potential of each interface classification mode.

As seen from Table 1, in the first four groups, the average Al–O ratios in the left and right interfaces are all less than 1, which we define as the O-dominated mode (O-dom). In the middle four groups, the average ratio of the interface is between 1 and 4/3, defined as the Al–O-balanced mode (Al–O-bal). In the last group, the average Al–O ratios of the interfaces are greater than 4/3, defined as the Al-dominated mode (Al-dom). Compared with the experimental results, the Al–O ratio in the O-dom indicates that the interface has a sub-oxide composition of Al, which is consistent with the conclusion of Vermeersch et al.^49^ through epitaxial growth of aluminum film and its oxide.

We calculate the potential of the nine sets of models in Table 1 one by one and find that the O-dom has the lowest average potential, followed by the Al-dom, and the Al–O-bal, as shown in Fig. 3. This shows the difference in transmission coefficients when electrons pass through the finite barrier formed by $\mathrm Al_2O_3$^38^. It is a reflection of difference in the potential barrier for electron tunneling. For systems with the same interface classification mode, the barrier height is uniformly estimated, and the distance between the first wave peak and trough in the right interface is used for comparison. The potential heights of the four models showing the O-dom in Fig. 3a are 1.93eV, 1.88eV, 1.74eV, and 1.95eV, respectively. The change in the potential height causes the change in the electron tunneling probability, which affects the transport properties of the system. The potential heights of O-dom is lower than that of the Al-dom and Al–O-bal, and the unbalanced height distribution of the left and right sides of the barrier may be due to the difference in the Al–O ratio between the left and right ones. Al-dom, which is mainly consistent of O-terminal (O–O) mode (as shown in Table 1), may attract Al atoms in the interface towards the barrier layers. The oxygen dangling bonds in the barrier have a greater probability of bonding with Al atoms in the interface, which will result in a lower conductance. In contrast, O-dom, which is mainly composed of 2Al terminal models, may form metallic channels, enabling electrons to pass through. The influence of the Al–O ratio of the interface on electrical properties will be further discussed in the following sections.

Figure 3d shows the change in the average barrier for different interface classification modes. The error bars show the effect of different oxide thicknesses on the potential height under the same mode. In the same interface classification mode, the change in oxide thickness has a subtle impact on the barrier height. On the contrary, the change in barrier height is more obvious in different modes. The difference in the electric potential inevitably leads to an internal additional electric field within the system, thereby promoting electron tunneling. Therefore, in the systems of the O-dom, the conductance is larger than that of the Al-dom and Al–O bal. In addition to the effect of thickness on the electrical properties of the junction, the Al–O ratio of the interface also contributes greatly to the properties of the junction.

### Comparative analysis of chips

**Figure 4:**
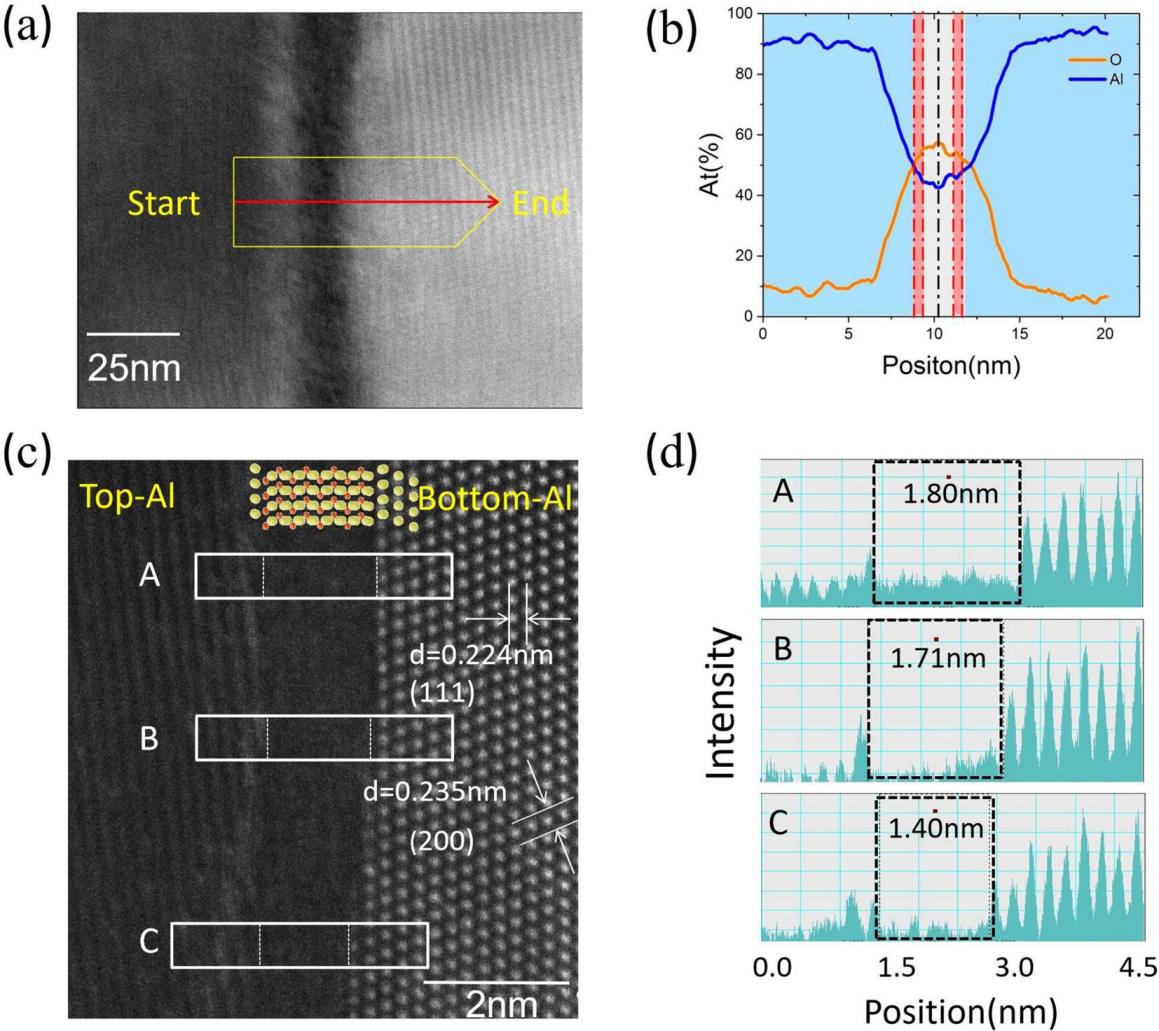
Structure of an Al/$\mathrm AlO_x$ /Al junction from STEM imaging and EDX analysis. (**a**) The red arrow area shows the EDX line scan area. (**b**) EDX line scan result. The red shading is the interface region. (**c**) A high resolution high-angle annular dark-field (HAADF) STEM image of the junction. (**d**) Image intensity profiles acquired from area A, B and C that are marked in (**c**).

The correspondence of the mode between the model and the JJ in the chip can be confirmed by high-resolution scanning transmission electron microscope (STEM) and energy dispersive X-ray spectroscopy (EDX) analysis. The EDX line scan results of the sandwich structure are shown in Fig. 4b, corresponding to the positions in the JJ in the red line area in Fig. 4a, to obtain the Al and O content distribution of JJ. It shows that the thickness of the oxide layer is about 2 nm, and the O content in the oxide layer is 55 At.%, consistent with the results of the molecular dynamics models^43,45^. The average thickness of the barrier and its variation are measured by selecting the intensity profile at different positions of the TEM. The measured positions are shown in Fig. 4c, with a window size of 0.6 nm $\times$ 3.5 nm, and their relative intensity variations are shown in Fig. 4d. The spikes of Al atoms at the bottom are relatively prominent, while the spikes of Al at the top are less obvious in Fig. 4d. The thickness of the intermediate $\mathrm AlO_x$ barrier layer is determined by distance comparison. As shown in Fig. 4d, the average thickness of the barrier layer is about 1.7 nm, consistent with the results obtained by EDX.

We conducted the resistance test in the chip at room temperature, with the resistance of 12k$\Omega$–14k$\Omega$. From the line scans of EDX, the Al:O of the interface is less than 1, which corresponds to the Al–O ratio of the O-dom in the model, as shown in Table 1. According to the conductance obtained by the model, it is converted into the resistance value under the same area, and the resistance value of the model is 14k$\Omega$–35k$\Omega$ in the thickness range, as shown in Table  2. The resistance of the test chip is consistent with the resistance range of the O-dom. Due to defects and other factors, the resistance value will fluctuate^50^.

**Table 2:** Comparison for electrical properties of models.

Interface classification	Atomic type of the interface	Thickness (Å)	Conductance (1E-6G0)	R (k$\Omega$)
O-dominated	Al–2Al	17.32	4.52	35.19
	2Al–Al	17.21	5.78	30.63
	O–2Al	16.60	5.66	31.29
	2Al–2Al	18.00	12.1	14.64
Al–O-balanced	Al-Al	16.42	4.19	42.3
	O–Al	17.98	0.41	427.84
	Al–O	18.00	0.56	316.56
	2Al–O	16.65	2.53	69.87
Al-dominated	O–O	17.71	0.42	418.73

For our desired qubit frequency, the normal state resistance is generally around 10k$\Omega$. Manufacturing other mode of junctions requires decreasing the barrier layer’s thickness to satisfy resistance specifications, as shown in Table 2. This may increase difficulty of the fabrication process. Due to the process error, we believe that there should be more than one termination mode in the actual chip, and finding a consistent process to prepare the termination mode is a way to obtain a flatter interface layer. However, there is currently no fabrication method to do like this. Finding other ways to improve electrical properties is another way. Considering the influence of the interface layer on the overall electrical properties, we further investigate the Al–O ratio of the interface.

### Aluminum oxidation stoichiometry

**Figure 5:**
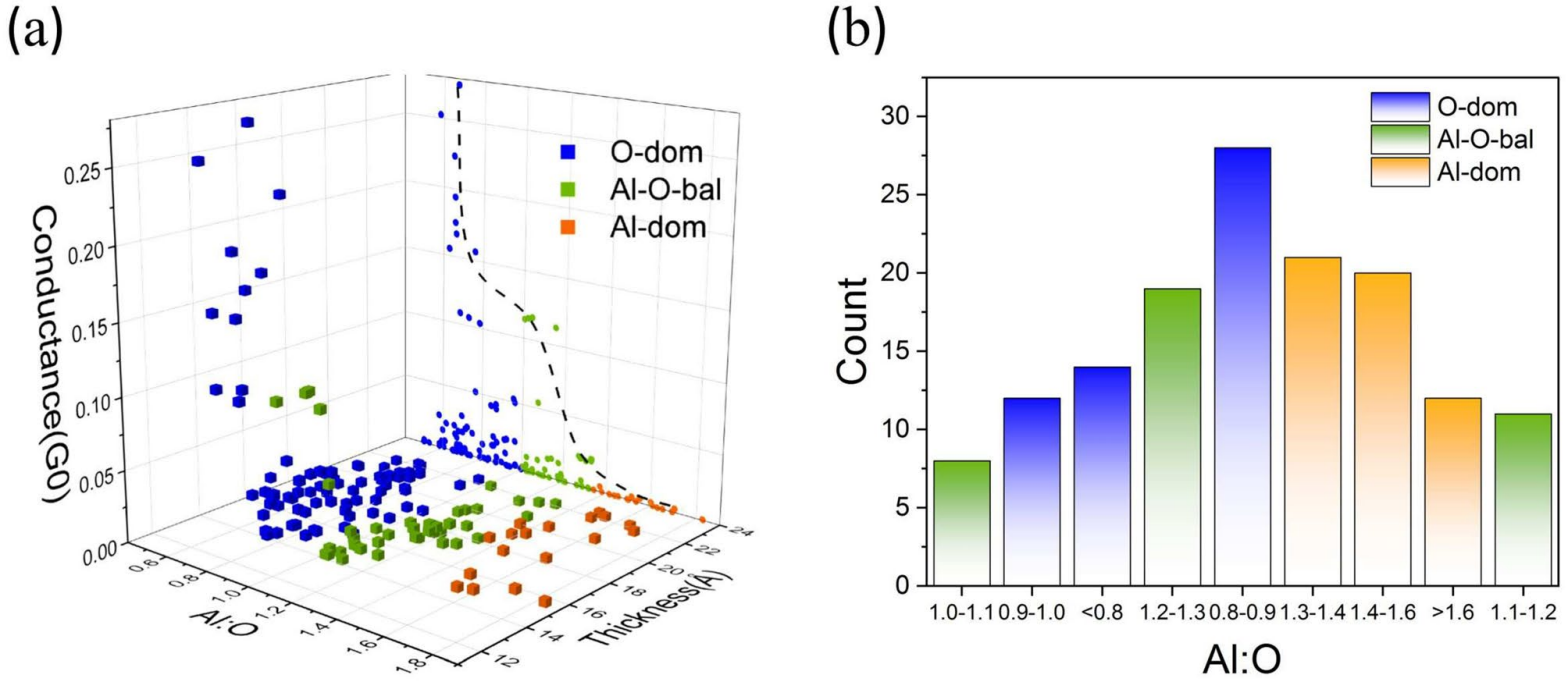
Statistical results of the conductance value and the thickness of the barrier layer, the distribution of Al–O ratio in the interface by utilizing GAN for data augmentation. (**a**) Diagram of Al–O ratio and conductance in the interface under different barrier thicknesses by using data augmentation model. The projection of XZ plane shows the Al–O ratio corresponding to the best conductance (also evident from the direction of the dashed black line). (**b**) shows statistical results of Al–O ratio by using data augmentation model.

By calculating the electrical properties of models, when the cross-sectional area of the barrier is fixed, the relationship among the Al–O ratio, the thickness and the conductance in the interface is analyzed. Due to the small amount of data, the regularity is difficult to find. GAN is used to expand the original samples. The expansion show that the conductance is still exponentially dependent on the thickness of the barrier, regardless of the classification mode of the interface, as shown in the projection of XZ plane of Fig. 5a. With the increase of the thickness, the conductance decreases exponentially^13^, which also verifies the credibility of the augmented data. As seen in Fig. 5a, when the thickness of the barrier is greater than 1.5 nm, the conductance changes slightly. When the barrier layer thickness is less than 1.5 nm, the conductance changes dramatically, considering that the contribution of the Al–O ratio is larger when the thickness is small in the barrier.

The relationship between the Al–O ratio and the conductance value in the interface is analyzed for different thicknesses, as shown in Fig. 5a. When the Al–O ratio is between 0.67 and 1.1, the electrical properties of JJ are the best, as shown from the projection of the XZ plane in Fig. 5a. As the thickness increases, the peak distribution of the Al–O ratio tends to decrease. From the statistical results, as shown in Fig. 5b, the distribution of the Al–O ratio is the highest when it is between 0.8–0.9. This is close to the average Al–O ratio (0.79) of the standard alumina. From the analysis of the barrier height, this average ratio is consistent with the O-dom with low barrier height. And from the energy, this ratio is also the most stable stoichiometry of $\mathrm Al_2O_3$^51^.

Overall, the electrical properties of JJ are affected by a various factors, including the thickness and the Al–O ratio. When the thickness is fixed, the Al–O ratio of the interface has a prominent influence on electrical properties. When the Al–O ratio of the interface is between 0.67 and 1.1, the maximum conductance is obtained. The statistical results show that the potential height is low, and the energy is most stable when the aluminum–oxygen ratio is between 0.8 and 0.9, resulting in the highest distribution.

## Conclusion

In summary, we propose a method for dividing the interface of JJ. This method can provide accurate and reasonable assistance in establishing a relationship between microstructure, macroscopic characterization and fabrication. Specifically, through this definition method, we can obtain the Al–O ratio of the interface in a region of the JJ in the chip through TEM and EDX, and verify the rationality of the definition method of the interface layer. NEGF and DFT are combined to calculate the conductance of Al/$\mathrm Al_2O_3$/Al device model, and the Al–O ratio and thickness data in the interface are statistically analyzed. We find when the thickness is fixed, the Al–O ratio of the interface greatly affects the electrical properties of the junction region. By adjusting the Al–O ratio in the fabrication process, we construct a bridge between microstructure and macroscopic characteristics using the key interface as the convergence point, and screen out factors that affect electrical properties except for thickness and junction area. The results will lay the groundwork for investigating new mechanisms of decoherence. Additionally, these provide theoretical guidance for optimizing process parameters, guiding the fabrication, and promoting the process iteration.

### Supplementary Information

Supplementary Information.

## Data Availability

The data that support the findings of this study are available from the corresponding author upon reasonable request.
