# Supplementary material for: Blurred interface induced control of electrical transport properties in Josephson junctions
Source: Sci Rep. 2024 Jul 27;14:17292. doi: 10.1038/s41598-024-68285-y (PMC11283497; doi:10.1038/s41598-024-68285-y)
Supplement: Supplementary file 1 — Supplementary Information. [file 41598_2024_68285_MOESM1_ESM.docx]

**Supplementary Information**

**Blurred interface induced control of electrical transport properties in Josephson junctions**

**Junling Qiu^1^, Huihui Sun^†1^, Chuanbing Han^1^, Xiaodong Ding^1^, Bo Zhao^1^, Shuya Wang^1^,Lixin Wang^1^, and Zheng Shan*^1^
^1^**Laboratory for Advanced Computing and Intelligence Engineering, Zhengzhou, 450001, China **Corresponding author**

*Zheng Shan

Email: shanzhengzz@163.com

^†^Huihui Sun

Email:sun_hui_hui@163.com

**EDX analysis of standard Al_2_O_3_**

To obtain the ratio of Al_2_O_3_ standard samples, we analyze the structural characteristics of pure Al_2_O_3_ liquid after dilution with propylene solution. From selected area electron diffraction(SAED), as shown in Fig. S1(a), the sample is a polycrystal of Al_2_O_3_ nanoparticles, with the main crystal orientations as [400],[220],[311]. Particles of heterogeneous size are present with a large number of particles from HRTEM, as shown in Fig. S1(b). It may be due to insufficient dilution. Ten points are collected in four different regions of the samples, and the Al-O ratios are between 0.64 and 0.94, with an average atomic ratio of 0.79. Three of these sampling points are shown in Fig. S1(c), where points are taken at different thicknesses to obtain more accurate component proportions. Fig. S1(d) shows the Al-O ratio of the Al_2_O_3_ standard samples at eight different positions, where the abscissa is the X-ray energy and the ordinate is the count.

**Supplementary Figure 1:** Structure of Al_2_O_3_ standard samples. (a) Al_2_O_3_ standard samples by SAED. (b)HRTEM of Al_2_O_3_ standard samples. (c)Al_2_O_3_ standard samples by TEM with 100nm and three sampling positions. (d)EDX analysis plots at eight different locations.
